# Diagnostic imaging for chronic plantar heel pain: a systematic review and meta-analysis

**DOI:** 10.1186/1757-1146-4-S1-P40

**Published:** 2011-05-20

**Authors:** Andrew McMillan, Karl Landorf, Joanna Barrett, Hylton Menz, Adam Bird

**Affiliations:** 1 Department of Podiatry, La Trobe University, Melbourne, Victoria 3086, Australia; 2 Musculoskeletal Research Centre, La Trobe University, Melbourne, Victoria 3086, Australia

## Background

Chronic plantar heel pain (CPHP) is a generalised term used to describe a range of undifferentiated conditions affecting the plantar heel. Plantar fasciitis is reported as the most common cause and the terms are frequently used interchangeably in the literature. Diagnostic imaging has been used by many researchers and practitioners to investigate the involvement of specific anatomical structures in CPHP. These observations help to explain the underlying pathology of the disorder, and are of benefit in forming an accurate diagnosis and targeted treatment plan. The purpose of this systematic review was to investigate the diagnostic imaging features associated with CPHP, and evaluate study findings by meta-analysis where appropriate.

## Methods

Bibliographic databases including Medline, Embase, CINAHL, SportDiscus and The Cochrane Library were searched electronically on March 25, 2009. Eligible articles were required to report imaging findings in participants with CPHP unrelated to inflammatory arthritis, and to compare these findings with a control group. Methodological quality was evaluated by use of the Quality Index as described by Downs and Black. Meta-analysis of study data was conducted where appropriate.

## Results

Plantar fascia thickness as measured by ultrasonography was the most widely reported imaging feature. Meta-analysis revealed that the plantar fascia of CPHP participants was 2.16 mm thicker than control participants (95% CI = 1.60 to 2.71 mm, *P* < 0.001) and that CPHP participants were more likely to have plantar fascia thickness values greater than 4.0 mm (OR = 105.11, 95% CI = 3.09 to 3577.28, *P* = 0.01). CPHP participants were also more likely to show radiographic evidence of subcalcaneal spur than control participants (OR = 8.52, 95% CI = 4.08 to 17.77, *P* <0.001).

## Conclusions

This systematic review has identified 23 studies investigating the diagnostic imaging appearance of the plantar fascia and inferior calcaneum in people with CPHP. Analysis of these studies found that people with CPHP are likely to have a thickened plantar fascia with associated fluid collection, and that thickness values >4.0 mm are diagnostic of plantar fasciitis. Additionally, subcalcaneal spur formation is strongly associated with pain beneath the heel.

